# Insulin-like growth factor 2 and autophagy gene expression alteration arise as potential biomarkers in Parkinson’s disease

**DOI:** 10.1038/s41598-022-05941-1

**Published:** 2022-02-07

**Authors:** Denisse Sepúlveda, Felipe Grunenwald, Alvaro Vidal, Paulina Troncoso-Escudero, Marisol Cisternas-Olmedo, Roque Villagra, Pedro Vergara, Carlos Aguilera, Melissa Nassif, Rene L. Vidal

**Affiliations:** 1 Center for Integrative Biology, Faculty of Sciences, Universidad Mayor, Santiago, Chile; 2 Biomedical Neuroscience Institute, Faculty of Medicine, University of Chile, Santiago, Chile; 3 Center for Geroscience, Brain Health and Metabolism, Santiago, Chile; 4 Hospital Fuerza Aérea de Chile, Santiago, Chile; 5 Departmento de Ciencias Neurológicas Oriente, Facultad de Medicina, Universidad de Chile, Santiago, Chile; 6 Department of Statistics and Econometrics, Universidad Tecnológica Metropolitana, Santiago, Chile; 7 Escuela de Biotecnología, Facultad de Ciencias, Universidad Mayor, Santiago, Chile

**Keywords:** Parkinson's disease, Biomarkers, Predictive markers

## Abstract

Insulin-like growth factor 2 (IGF2) and autophagy-related genes have been proposed as biomolecules of interest related to idiopathic Parkinson’s disease (PD). The objective of this study was to determine the IGF2 and IGF1 levels in plasma and peripheral blood mononuclear cells (PBMCs) from patients with moderately advanced PD and explore the potential correlation with autophagy-related genes in the same blood samples. IGF1 and IGF2 levels in patients' plasma were measured by ELISA, and the IGF2 expression levels were determined by real-time PCR and Western blot in PBMCs. The expression of autophagy-related genes was evaluated by real-time PCR. The results show a significant decrease in IGF2 plasma levels in PD patients compared with a healthy control group. We also report a dramatic decrease in *IGF2* mRNA and protein levels in PBMCs from PD patients. In addition, we observed a downregulation of key components of the initial stages of the autophagy process. Although IGF2 levels were not directly correlated with disease severity, we found a correlation between its levels and autophagy gene profile expression in a sex-dependent pattern from the same samples. To further explore this correlation, we treated mice macrophages cell culture with α-synuclein and IGF2. While α-synuclein treatment decreased levels *Atg5*, IGF2 treatment reverted these effects, increasing *Atg5* and *Beclin1* levels. Our results suggest a relationship between IGF2 levels and the autophagy process in PD and their potential application as multi-biomarkers to determine PD patients' stages of the disease.

## Introduction

Parkinson’s disease (PD) is one of the most frequent neurodegenerative diseases with a prevalence of 1% in persons over 60 years and 3% in people over 80 years^1^. There are an estimated 40,000 patients with PD in Chile^2^. PD is characterized by motor dysfunction with both cardinal symptoms such as bradykinesia, rigidity, tremor at rest^3^, and axial symptoms such as gait disturbances and postural instability^4,5^. Tremor at rest is the classic manifestation, with a 4–6 Hz frequency, but can be absent in 25% of the patients at diagnosis^3^. Additionally, non-motor symptoms in PD include smell disorders, constipation, sleep disturbances like rapid-eye-movement (REM) behavioral disorder, among others^6,7^, which are manifested in the early stages of the pathology. At the molecular level, the accumulation of the synaptic protein α-synuclein in Lewy bodies is a histopathological hallmark of idiopathic and familial PD cases^8,9^. The expression of α-synuclein is not limited to the nervous system but also is present in the cerebrospinal fluid (CSF), in plasma^10,11^, as well as being expressed in the erythropoietic lineage cells^12^ and peripheral lymphocytes^13^. However, the definitive diagnosis of PD is based on motor impairment when the neurodegenerative process is highly advanced. The finding of biomarkers for an accurate diagnosis and progression monitorization of PD remains unsolved.

An ever-increasing number of humoral growth factors active in different tissues have been implicated in brain physiology. Insulin and its related peptides, insulin-like growth factors 1 and 2 (IGF1 and IGF2), have been recognized as neuroactive peptides, influencing neuronal homeostasis through different mechanisms of action^14^. The IGF system comprises hormonal ligands, insulin, IGF1, and IGF2 and their receptors, IR, IGF1R, and IGF2R^15^. Studies in cellular and preclinical models of neurodegenerative diseases have validated the neuroprotective effects of IGFs to prevent disease progression^16^, including PD, particularly considering their roles as (i) neurogenic agents and (ii) their apparent neuroprotective effect^17–19^. In the case of the neurotrophic factor IGF1, it has a role in brain development, neuroprotection, and neurogenesis^20^. In PD, IGF1 has shown a neuroprotector effect, reducing apoptosis levels in the toxicity induced by dopamine in neuronal cultures^21^. Also, it reduces α-synuclein toxicity and protein aggregates formation in neuroblastoma cell lines^22^. In rat models of PD, IGF1 treatment has shown beneficial effects in preventing dopaminergic neuronal loss in the substantia nigra^23^ and improving motor deficits^24,25^. Accumulating evidence suggests that a progressive decline in the levels of serum IGF1 may also contribute to age-associated brain disorders^20,26,27^. Moreover, an increase in IGF1 levels has been reported in serum samples from PD patients suggesting a compensatory effect in this pathology^28–30^.

On the other hand, IGF2 is a single-chain polypeptide expressed in most tissues during fetal life and found predominantly within the brain and spinal cord during adulthood^31,32^. Several pieces of evidence highlight the physiological function of IGF2 in cognition, neuronal differentiation, and neuronal survival^33–37^. For instance, *Igf2* is a target of the transcription factor C/EBPβ that is engaged during memory consolidation in rats^38^, and IGF2 overexpression rescues working memory deficits in a mouse model of schizophrenia^39^. Also, IGF2 has been described as a regulator of adult hippocampal neurogenesis^40^ and a neuron survival factor in in vitro studies^41,42^. It has also been shown that IGF2 participates in neuromuscular synapse formation^43^. Interestingly, IGF2 expression is decreased in the hippocampus of Alzheimer’s disease (AD) patients and a mouse model of this disease^44^. The IGF2 overexpression reduced amyloid-β plaques in the hippocampus of transgenic AD mice^44,45^, which was mediated by the IGF2R^45^. Recently, we described the protective role of IGF2 in Huntington's disease (HD)^33^. IGF2 administration diminished the load of mutant huntingtin protein in HD preclinic models and iPSCs from HD patients. We determined a significant decrease in plasmatic IGF2 levels in blood samples from HD patients^33^. IGF2 also showed a substantial expression reduction in peripheral blood mononuclear cells (PBMCs) and brain tissues from HD patients, arising as an interesting biomarker for neurodegenerative diseases. Remarkably, genetic linkage studies have associated IGF2 with PD. A single-nucleotide polymorphism (SNP) in the *IGF2* gene was identified as a potential modifier of the susceptibility to develop idiopathic PD in a Caucasian group from Australia^46^. This polymorphism was associated with reduced IGF2 expression levels, lower body weight, and low levels of tyrosine hydroxylase enzyme (TH) expression, the limiting enzyme in dopamine production^47^. PD patients usually present a low body mass index, potentially reflecting a broader metabolic disorder^48,49^. IGF2 also participates in immune reaction regulation, promoting in macrophages an anti-inflammatory profile^50,51^. However, the molecular mechanism involved in IGF2 neuroprotection is still under study.

IGF2/IGF2R signaling participates in the transport of lysosomal hydrolases from the Golgi apparatus to lysosomes^52,53^, suggesting a potential connection between IGF2 with the autophagy-endolysosomal pathway. It has been demonstrated that IGF2 is overexpressed in colorectal cancer cells and that this phenomenon is associated with increased autophagy activity^54^. Moreover, the overexpression of IGF2 in pancreatic β cells is associated with increased ER stress, autophagy activation, and β cells dedifferentiation^55^. Macroautophagy (hereafter referred to as autophagy) is the primary intracellular process responsible for cargos' bulk or selective degradation by delivering them to lysosomes by vesicle traffic. This process is highly regulated by several ATG (autophagy-related genes) protein complexes^56^. The autophagy process is an important pathway for the clearance of misfolded proteins involved in neurodegenerative diseases such as tau, mutant huntingtin, ataxin-3, and mutated SOD1^57,58^. It has been described that selective chaperone-mediated autophagy (CMA) is involved in α-synuclein degradation in PD in vivo models and PD patients^59,60^.

Lysosomes and autophagosome vesicles were reported together with Lewy bodies and α-synuclein in postmortem PD brain samples^61^. The expression of autophagy genes was also explored in blood samples from moderate to advanced PD patients. A downregulation was reported in mRNA levels of six core regulators of autophagy *ULK1, ATG5, ATG2A, ATG4B, ATG6L1,* and *HDAC6* in PBMCs from PD patients^62^. Interestingly, protein levels of ULK1, BECLIN1, and AMBRA1, proteins participating at initial steps of the pathway, were increased in PBMCs samples obtained from PD patients^62^. Although some links have been proposed between IGFs and the lysosomal-autophagy pathway, IGF1 or IGF2 levels and the expression of autophagy genes in PBMCs from PD patients have not yet been associated. These data could contribute to a multi-biomarker approach to monitoring PD progression and diagnosis.

In the present work, we evaluated IGF1 and IGF2 levels and the transcriptional expression of key genes from the autophagy pathways in blood samples from male and female sporadic moderate to severe PD patients and their association with the pathology's severity. We observed a significant reduction in IGF2 mRNA and protein levels in PD patients instead of no differences in IGF1. We also found a decrease in *ATG5*, *ULK1*, and *BECLIN1* transcriptional levels in PBMCs samples from sporadic PD patients, associated with the reduction of IGF2 mRNA and protein levels. To assess the effect of IGF2 on autophagy genes expression in a PD context, we treated primary macrophages with IGF2 after preincubation with α-synuclein preformed fibrils. IGF2 treatment reversed the downregulation of *Atg5* and *Beclin1* caused by α-synuclein, indicating a potential IGF2 positive effect on autophagy activity in a PD model. Our results show decreased plasma IGF2 levels as an easily reached outcome for PD. It is associated with autophagy genes levels, particularly from the initial steps of autophagosome formation.

## Materials and methods

### Study subjects

All participants, 41 healthy controls and 43 PD patients, were recruited and signed written informed consents. The diagnosis of PD patients was based on clinical history, physical examination, and a score from the Hoehn and Yahr modified^63^ and UPDRS^64,65^ scale. Healthy controls (HC) were submitted to the same neurological and neuromotor evaluations. This study followed the Declaration of Helsinki and was approved by the local Ethical Committee of the FACH hospital (FACH Human Subject Protocol Number #181004). As inclusion criteria, we considered subjects over 18 years old, having a history of attention in Hospital Clínico Fuerza Aérea de Chile (FACH) and PD patients in stages 1–4 of the Höehn and Yahr modified scale. Exclusion criteria included patients under 18 years of age and subjects diagnosed with PD in stage 5 of the Höehn and Yahr modified scale. Tables 1 and 2 show the demographic data of the study participants. The average medical history of PD patients is 6.6 ± 5.6 years. Evaluation with the Höehn and Yahr scale showed the majority of PD patients in the cohort were in stage 3 (58%), and 92–95% of the patients had a mild to moderate state of disease (between 1 to 3 of the Höehn and Yahr scale) which corresponds to a state of self-balance in patients with postural stability disorder. The average motor UPDRS was 34.6 ± 12.5, categorizing the patients in an intermediate state of the disease concerning their motor condition. There was no significant difference in participants' age or gender ratio in both groups (PD and HC).

**Table 1:** Demographics and clinical characteristics of Parkinson’s disease patients (PD) and healthy controls (HC).

	PD (n = 43)	HC (n = 41)	P value
Age (years)	71.2 ± 7.87	67.5 ± 7.82	No significance
Gender (male/female)	22/21	17/24	No significance
Years of disease	6.6 ± 5.6	–	
Hoehn and Yahr stage	3 (1–4)	–	
UPDRS-III score	34.6 ± 12.5	–

**Table 2:** Distribution by Hoehn and Yahr stage for Parkinson’s disease (PD) patients.

Hoehn and Yahr stage	Number of patients	Percentage
1	1	2.3%
2	14	32.5%
3	25	58.1%
4	3	6.9%

A normality test was achieved, for the ages of both groups, using the Shapiro-Wilks test^107^, obtaining normality in both controls subjects (SW-W = 0.955, p = 0.1052) and PD patients (SW-W = 0.9767, p = 0.5227). A test for equality of variances was performed, resulting in both being equal. A t-Student test showed no difference between patients and controls. A χ^2^ test was performed, resulting in no difference between genders.

### Plasma and PBMCs isolation

The extraction of PBMCs was performed from peripheral blood collected (30 mL) in sodium heparin vacutainer tubes at the FACH Hospital, according to our previous report^66^. Blood samples were taken between 10:00 and 12:00 AM in non-fasting patients. In brief, cells were separated from whole blood by Ficoll-Hypaque density centrifugation, and mononuclear cells (Lymphocytes-Monocytes) were rescued^33,67^. Three million cells were preserved in 1 ml of TriZol (Ambion) and stored at -80 °C for mRNA extraction or homogenized for Western blot (below). The plasma fraction was divided into aliquots to avoid cycles of freezing and thawing prior to ELISA measurements.

### Western blot and ELISA

For Western blot analysis, PBMCs were homogenized in RIPA buffer (20 mM Tris pH 8.0, 150 mM NaCl, 0.1% SDS, 0.5% Triton X-100) containing protease and phosphatase inhibitors (Roche). After sonication, protein concentration was determined in all experiments by micro-BCA assay (Pierce), and 20–30 µg of total protein was loaded onto 8 to 15% SDS-PAGE mini gels (Bio-Rad Laboratories, Hercules, CA) prior to transfer onto PVDF membranes. Membranes were blocked using PBS, 0.1% Tween-20 (PBST) containing 5% milk for 60 min at room temperature and then incubated overnight with primary antibodies in PBS, 0.02% Tween-20 (PBST) containing 5% skimmed milk. The following primary antibodies and dilutions were used: anti-IGF2 1:1000 (Abcam, Cat. nº Ab9574), anti-GAPDH 1:2000 (Santa Cruz, Cat. nº SC-365062). Bound antibodies were detected with peroxidase-coupled secondary antibodies incubated for 2 h at room temperature and the ECL system. The plasma levels of IGF1 and IGF2 were measured by ELISA (ALPCO, 22-IGFHU-E01, and 22-IG2HU-E01) according to the manufacturer’s instructions.

### Bone marrow macrophages isolation

α-synuclein overexpressing (ASO) mice at 3 months old^68^ were euthanized by isoflurane overdose. Bone marrows from the femur cavity were extracted as previously described^69^. In brief, bone marrows were extracted with a needle using a sterile 15-ml tube. Cells were centrifuged for 5 min at 500×*g*, at room temperature. Cell pellets containing macrophages were resuspended with complete medium (MCS-F) by tapping the tube, pipetting up and down, then transferring them to a 100 mm sterile plate. Macrophages were maintained at 37 °C, 5% CO_2_ incubator for seven days. To determine the effect of IGF2 on the autophagy genes expression, macrophages were treated with recombinant IGF2 (5 ng/mL, Sigma) for 1, 3, and 5 days^50^. After the IGF2 treatment, macrophages were stimulated with 1 µg of recombinant mouse α-synuclein PFFs were generated as previously described^70^ or PBS for 72 h.

### Human PBMCs extraction and mice macrophage gene expression analysis

Total mRNA was isolated using the TriZol reagent (Thermo Fisher). A DNAase digestion step with TURBO DNA-free TM Kit was included to remove any genomic DNA contamination. The 260/280-absorbance ratio assessed RNA purity. The quality and quantity of RNA in all participants were confirmed. cDNA was synthesized from total RNA (1 μg) using the High Capacity cDNA Reverse Transcription Kit (Thermo Fisher) in the T100TM Thermal Cycler (Bio-Rad). Real-time quantitative PCR (qPCR) was performed in an amplification system CFX96 TM Real-time System, using the DNA binding dye SYBR Green (Bio-Rad). A CFX96 real-time PCR detection system (Bio-Rad) was used to assess the mRNA levels of IGF2 and autophagy-related mRNA levels using the following human primers: *IGF2*: 5’- GTG CTG TTT CCG CAG CTG-3’ and 5’- AGG GGT CGA CAC GTC CCT C-3’ *ULK-1*: 5’- CAG ACG ACT TCG TCA TGG TC-3’ and 5’-AGC TCC CAC TGC ACA TCA G-3’, *P62*: 5’-TGC CCA GAC TAC GAC TTG TG-3’ and 5’- AGT GTC CGT GTT TCA CCT TCC-3’, *LC3*: 5’-CAT GAG CGA GTT GGT CAA GA-3’ and 5’-CCA TGC TGT GCT GGT TCA-3’, *RUBICON*: 5’-CTG GCA GTT CGT GAA AGA CA- 3’ and 5’-TTA GCA GGA AGG CAG CAT CT-3’, *ATG5*: 5’-TTT GCA TCA CCT CTG CTT TC-3’ and 5’-TAG GCC AAA GGT TTC AGC TT-3’, *BECLIN1*: 5’-GGA TGG TGT CTC TCG CAG AT-3’and 5’-TTG GCA CTT TCT GTG GAC AT-3’; and, as housekeeping genes, *SDHA*: 5’-GAG GCA GGG TTT AAT ACA GCA-3’and 5’-CCA CCA CTG GGT ATT GAG TAG AA-3’, and, when pointed, *ACTIN*: 5’-GCG AGA AGA TGA CCC AGA TC-3’ and 5’- CCA GTG GTA CGG CCA GAG G-3’. The murine primers used were as follows: *mATG5*: 5’-GCC TAT ATG TAC TGC TTC ATC CA-3’ and 5’-CAT TTC AGG GGT GTG CCT TCA-3’; *mBeclin1:* 5’-GTG CGC TAC GCC CAG ATC-3’ and 5’-GAT GTG GAA GGT GGC ATT GAA-3’; *mLC3*: 5’-CGG AGC TTT GAA CAA AGA GTC-3’ and 5’-TCT CTC ACT CTC GTA CAC TTC-3’; *mp62:* 5’-TGA AAC ATG GAC ACT TTG GCT-3’ and 5’-ACA TTG GGA TCT TCT GGT GGA-3’; and, as a housekeeping gene, *mActin1*: 5’-GGC TGT ATT CCC CTC CAT CG-3’ and 5’-CCA GTT GGT AAC AAT GCC ATG T-3’. RT-PCR conditions were: 1 cycle at 95 °C for 3 min, followed by 40 cycles at 95 °C for 30 s. Transcript levels were quantified using the ΔCt value method, mRNA levels of interesting genes were quantified and normalized to housekeeping mRNA levels. Additionally, the raw data were normalized to a value of 1 related to the healthy control group.

### Statistical analysis

The statistical analysis used to compare the expression of each of the IGF2/IGF1 and autophagy genes between the different groups was performed using the Mann–Whitney test after the Shapiro–Wilk test confirmation of non-parametric samples. An outliers test was performed in each measurement, resulting in differences in sample numbers in each experiment. We used the outliers test from Prism 9 software to apply the ROUT method and consider Q = 1%. This method is based on FDR, and the Q value is similar to alpha. Pearson statistical analysis was performed to correlate plasma levels of IGF1 and 2 with the clinical stage of PD. The Mann–Whitney test was also performed to analyze the differences between controls and patient groups for protein levels (non-parametric). A heat map and a dendrogram analysis were performed using the GraphPad Prism 9 and Statistics 10.0 software to evaluate the potential clustering of patients and controls based on the expression levels of IGF2 and autophagy genes evaluated. To potentially categorize a predominant behavior for each patient based on the evaluated variables, IGF2 and autophagy genes expression, we performed a principal component analysis (PCA), a statistical method used to predict responses based on a group of multiple variables' information^71–73^ using the Statistics 10.0 software^74^. mRNA expression levels in macrophage cell culture were analyzed using ordinary one-way ANOVA (parametric data). N indicates the number of biological replicates. On all graphs, error bars represent SEM (standard error for the media). GraphPad Prism software 9.0 was used for statistical analysis.

## Results

### IGF2 is downregulated in the blood of PD patients

**Figure 1:**
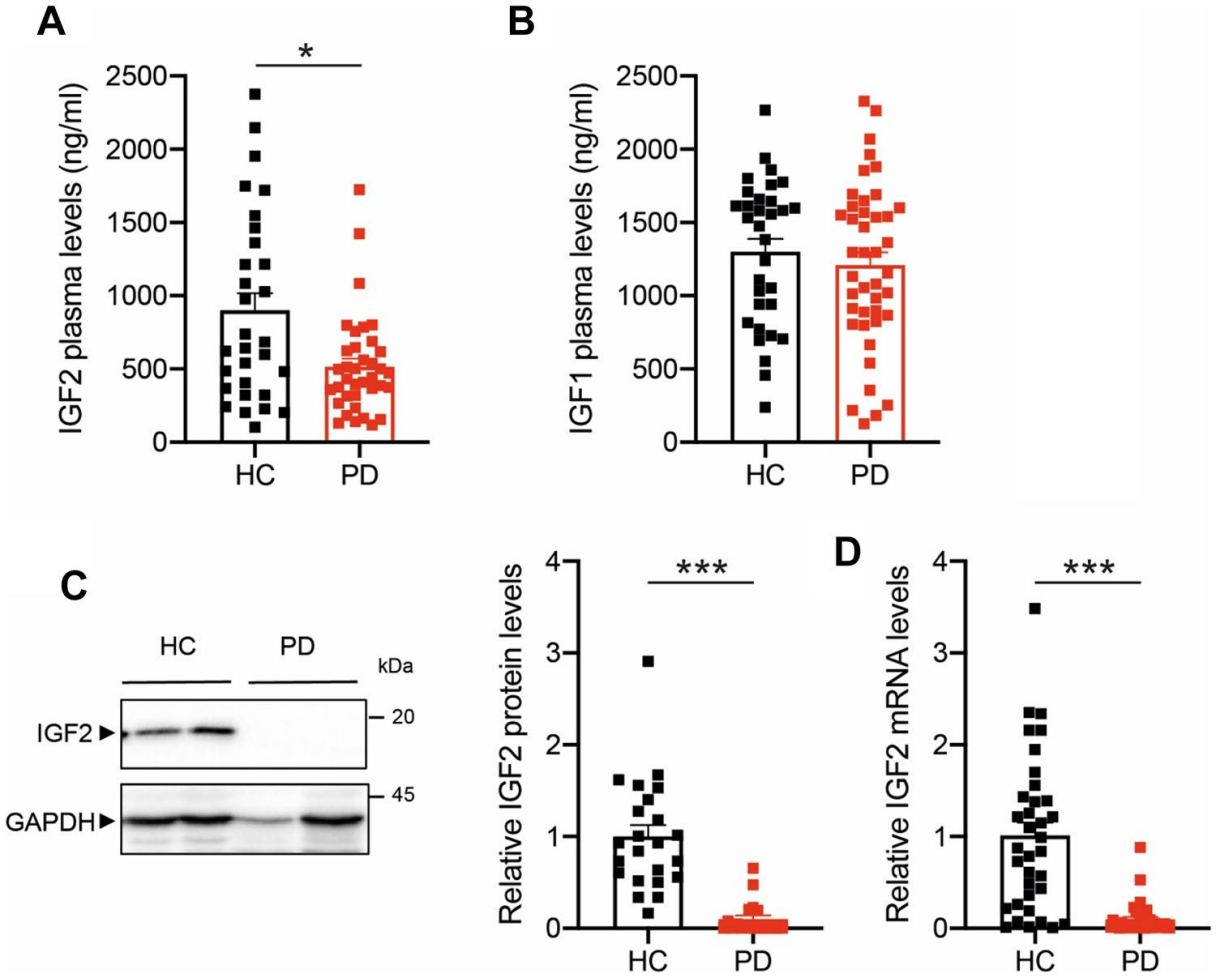
The IGF2 is downregulated in the blood of PD patients. (**A**,**B**) IGF1 and IGF2 content in plasma obtained from PD patients and healthy control (HC) subjects were measured by ELISA. (**C**) Representative IGF2 WB of total protein extracts isolated from peripheral blood mononuclear cells (PBMCs) of PD patients and HC subjects. GADPH was determined as a loading control (left panel). IGF2 levels were quantified and normalized to GADPH levels (right panel). (**D**) *IGF2* mRNA levels were measured and normalized to *SHDA* mRNA levels by real-time PCR in PBMCs obtained from PD patients and HC subjects. (PD, N = 40; HC, N = 40). Statistically significant differences were detected by two-tailed unpaired *t*-test (***p < 0.001; *p < 0.05).

**Figure 2:**
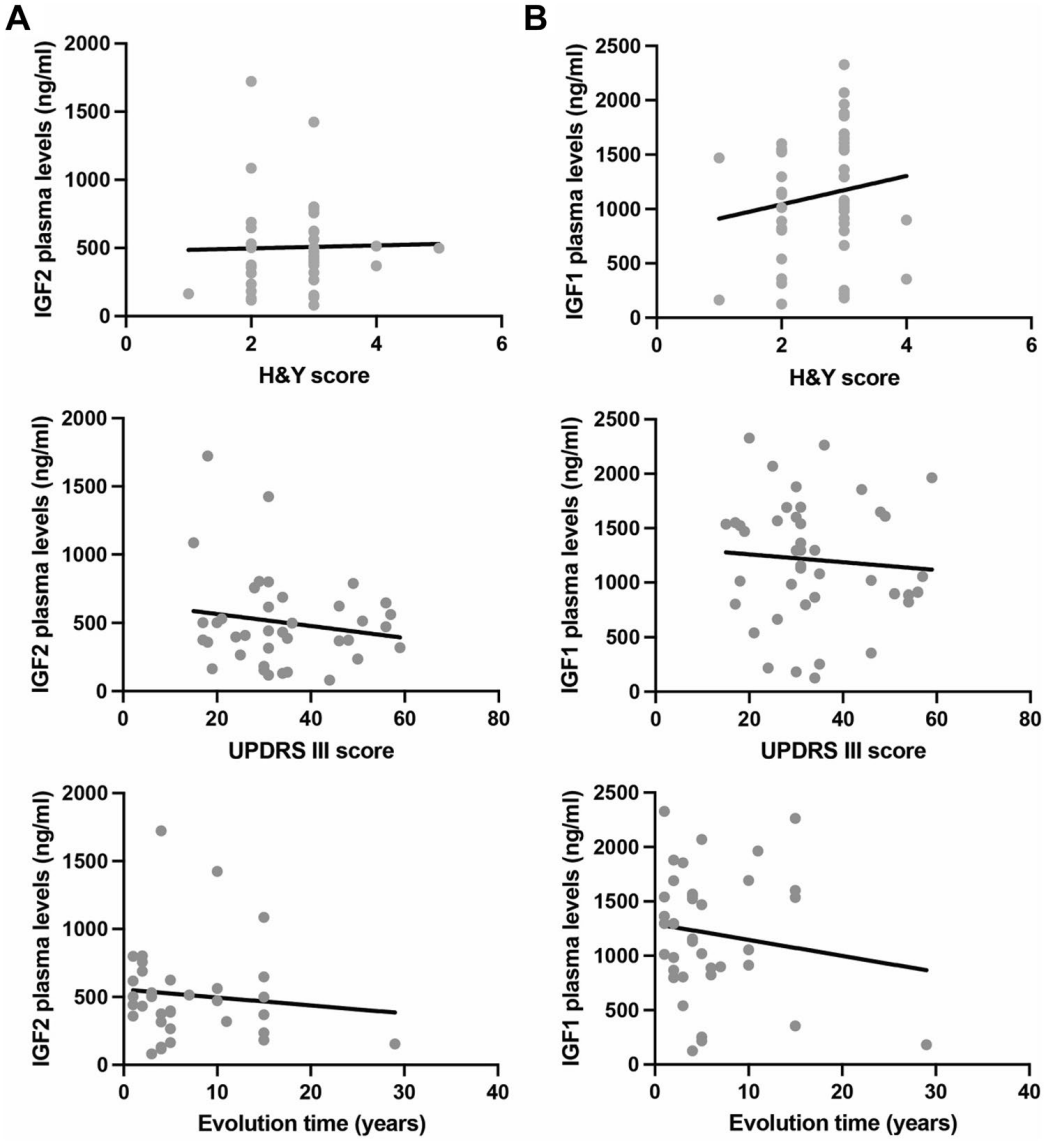
Plasma IGF1 and IGF2 levels in PD patients do not correlate with motor scores of PD patients. (**A**) Pearson correlation analysis between IGF2 levels in PD patients with H&Y (top panel, r = 0.1371), UPDRSIII (middle panel, r = − 0.07482) scores and evolution disease time (bottom panel, r = − 0.1951). (**B**) Correlation analysis between IGF1 levels in PD patients with H&Y (top panel, r = 0.1498), UPDRSIII (middle panel r = − 0.07823) scores and evolution disease time (bottom panel, r = − 0.09742).

Different studies suggest that changes in cellular homeostasis in the brain can be reflected in variations in the concentration of different plasma components, such as secreted factors or even changes in the gene expression profile. Since IGF2 is a soluble secreted factor, we measured IGF2 in plasma from PD patients using ELISA. This analysis revealed a slight but significant decrease in circulating IGF2 present in the plasma samples derived from PD patients compared to control subjects (p = 0.0185, Fig. 1A), which may be related to the different contributions of tissues and cell types to plasmatic IGF2 levels. However, the soluble IGF1 plasma levels were similar in PD patients compared to healthy control (HC) (p = 0.4186, Fig. 1B). We then evaluated the expression of IGF2 in PBMCs from HC and PD patients. Although control PMBCs presented a clear protein band for IGF2 by Western blot, PD-derived cells had a significant decrease in IGF2 levels, showing a nearly 70% decrease in its protein levels using Western blot analysis (p < 0.0001, Fig. 1C and supplementary Fig. S1). These results were confirmed when mRNA levels of *IGF2* were measured in the same samples, showing a near 90% decrease in its expression (p < 0.0001, Fig. 1D and supplementary Fig. S2). Taken together, these results suggest that IGF2 levels are drastically reduced in plasma and blood cells of PD patients. Demographic and clinical characteristics of PD patients and HC are shown in Table 1. However, plasma IGF1 or IGF2 levels were not correlated with clinical scores (UPDRSIII and H&Y) in PD patients (Fig. 2).

### Autophagy components are altered in PBMCs from PD patients

**Figure 3:**
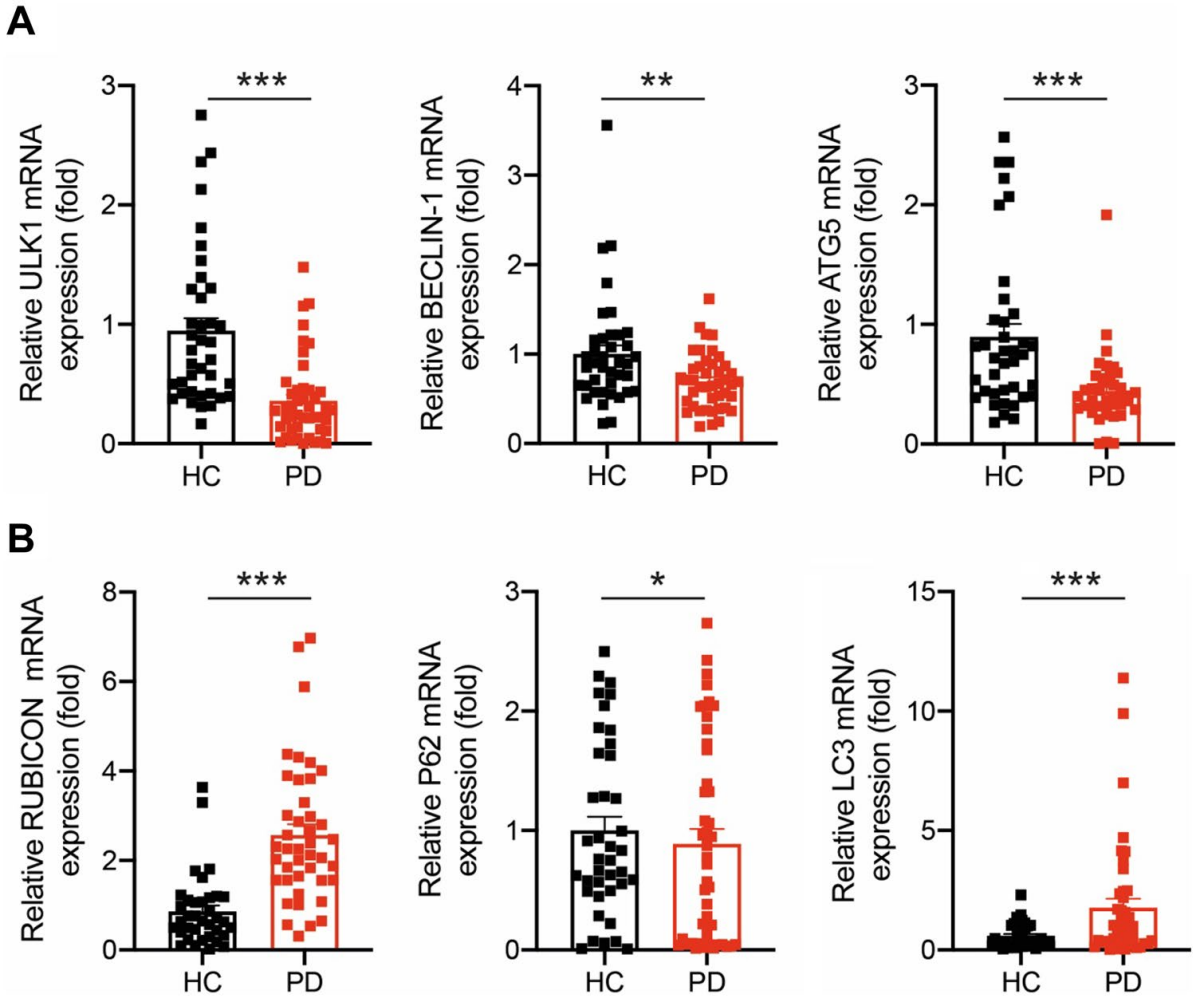
Autophagy components are altered in PBMCs from PD patients. (**A**,**B**) mRNA levels were measured by real-time PCR in PBMCs obtained from PD patients and HC subjects. (PD, N = 40; HC, N = 40). Autophagy-related gene mRNA levels were quantified and normalized to *SHDA* mRNA levels. Statistically significant differences were detected by two-tailed unpaired *t*-test (***p < 0.001; *p < 0.05).

Considering previous reports describing a positive correlation between IGF2 and the autophagy pathway in different tissues^54,55^, we determined the levels of autophagy-related genes in PBMCs from PD patients and HC. We observed a significant downregulation of *ATG5*, *ULK1*, and *BECLIN1,* genes involved at initial stages in the autophagy pathway, in PD patients compared to HC (Fig. 3A). Furthermore, we observed an upregulation of *RUBICON* and *LC3B* mRNA levels in PD patients compared to HCs (Fig. 3B). However, we did not observe significant changes in *P62* levels, a selective autophagy receptor (Fig. 3B).

**Figure 4:**
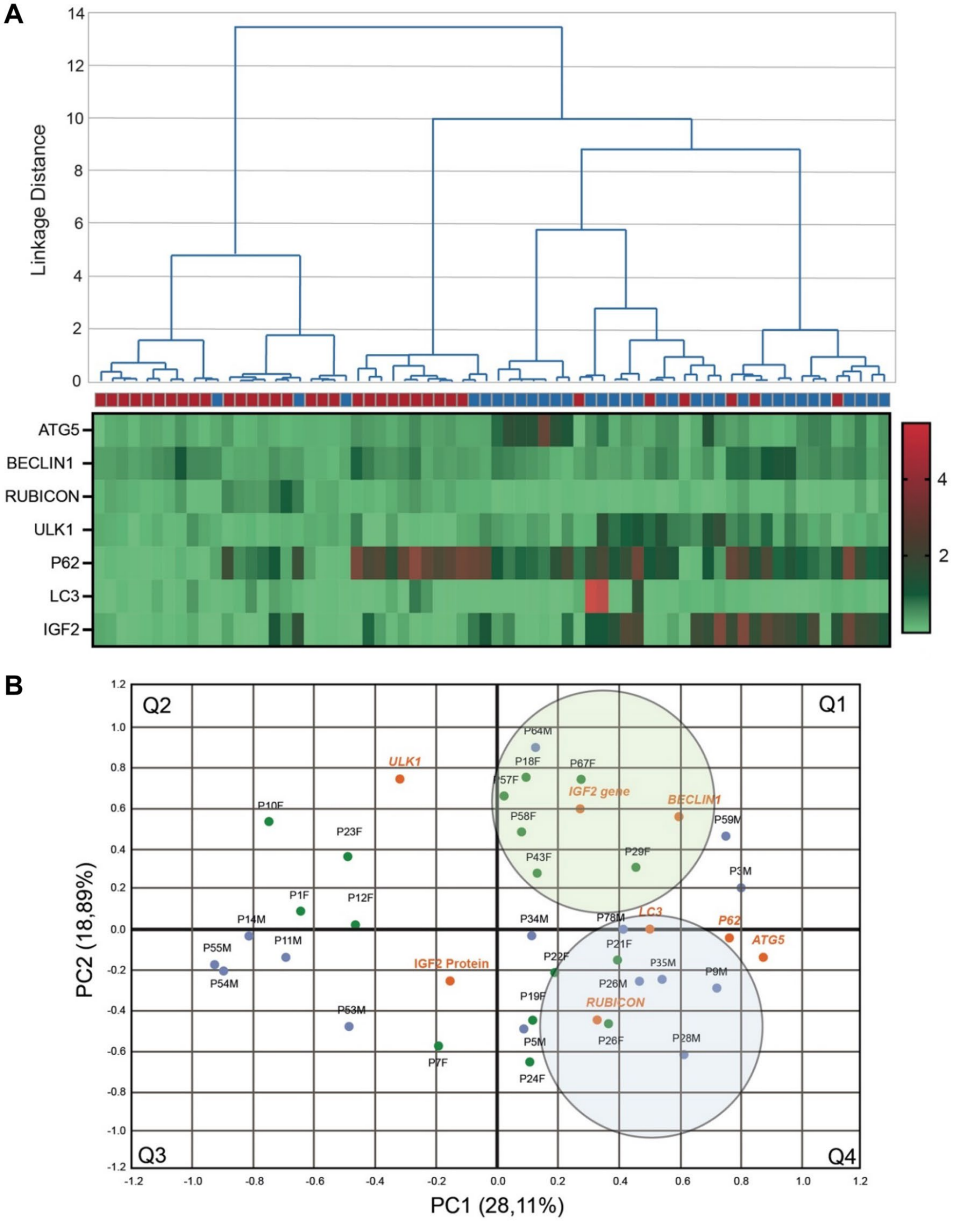
Analysis of IGF2 and autophagy markers of Parkinson ‘s disease patients and healthy controls. **(A)** Dendrogram and Heat Map analysis of gene expression patterns measured in PBMCs from Parkinson's disease patients (red squares) and Healthy Controls (blue squares). **(B)** Principal component analysis (PCA) considers all variable measures in blood samples (PMBCs and plasma) from PD patients distributed in four quadrants (Q1–Q4) depending on their expression profile. Orange dot represent the variables measured in blood samples from PD patients. Female patients are represented in green dots. Male patients are represented in blue dots. (N = 33/38 HC/PD). The large circles encompass patients with a similar expression profile, which diameters have no relationship with the statistical significance.

We generated a dendrogram analysis and heat map to determine each autophagy component and IGF2 levels' correlation with the different subjects in the HC and PD groups. We observed a clear difference in the gene expression profile between the PD group (Fig. 4A, red squares) and HC group (Fig. 4A, blue squares), showing a decrease in the *IGF2* levels and the expression of genes from the initial step of the autophagy pathway, excepting *LC3B* and *RUBICON*. Next, we evaluated the most predominantly variables (IGF2 and autophagy gene expression) associated with PD patients using a PCA, a statistical method that allows the association of the profile of gene expression for each patient^71–73^. For instance, we can find an association of patients' gender with a specific gene expression profile using this method. Patients were organized into four quadrants depending on IGF2 levels (IGF2 protein and gene expression) and autophagy genes transcriptional expression. This analysis revealed a clear separation profile for two groups of patients (PC1 and PC2). The variance of the PC1 represents 28.11% of the variation among patients, and the variance of the PC2 represents 18.89% within the data (Fig. 4B). The PCA analysis showed that the variable most predominant in patients situated in quadrant 1 (Q1) is *IGF2* and *BECLIN1* expression; patients in quadrant 2 (Q2), are associated with *ULK1*; and patients situated in quadrant 4 (Q4) have in common *ATG5* levels as the most predominant variable (Fig. 4B). Patients situated in Q3, however, did not show significant association with the variables evaluated. Notably, patients in Q1 who strongly correlated with lower *IGF2* and *BECLIN1* gene expression are most represented by female patients (green circles). Alternatively, male patients were more related to reduced expression of the autophagy gene *ATG5* and increased *LC3B* and *RUBICON* (blue circles) in Q4 (Fig. 4B and supplementary Fig. S3). Overall, these results suggest a distinct expression profile in autophagy genes expression between female and male PD patients. Moreover, *IGF2* transcriptional levels in PD patients were correlated with reduced levels of *BECLIN1*, especially in females.

### IGF2 treatment increases autophagy genes expression in macrophages

**Figure 5:**
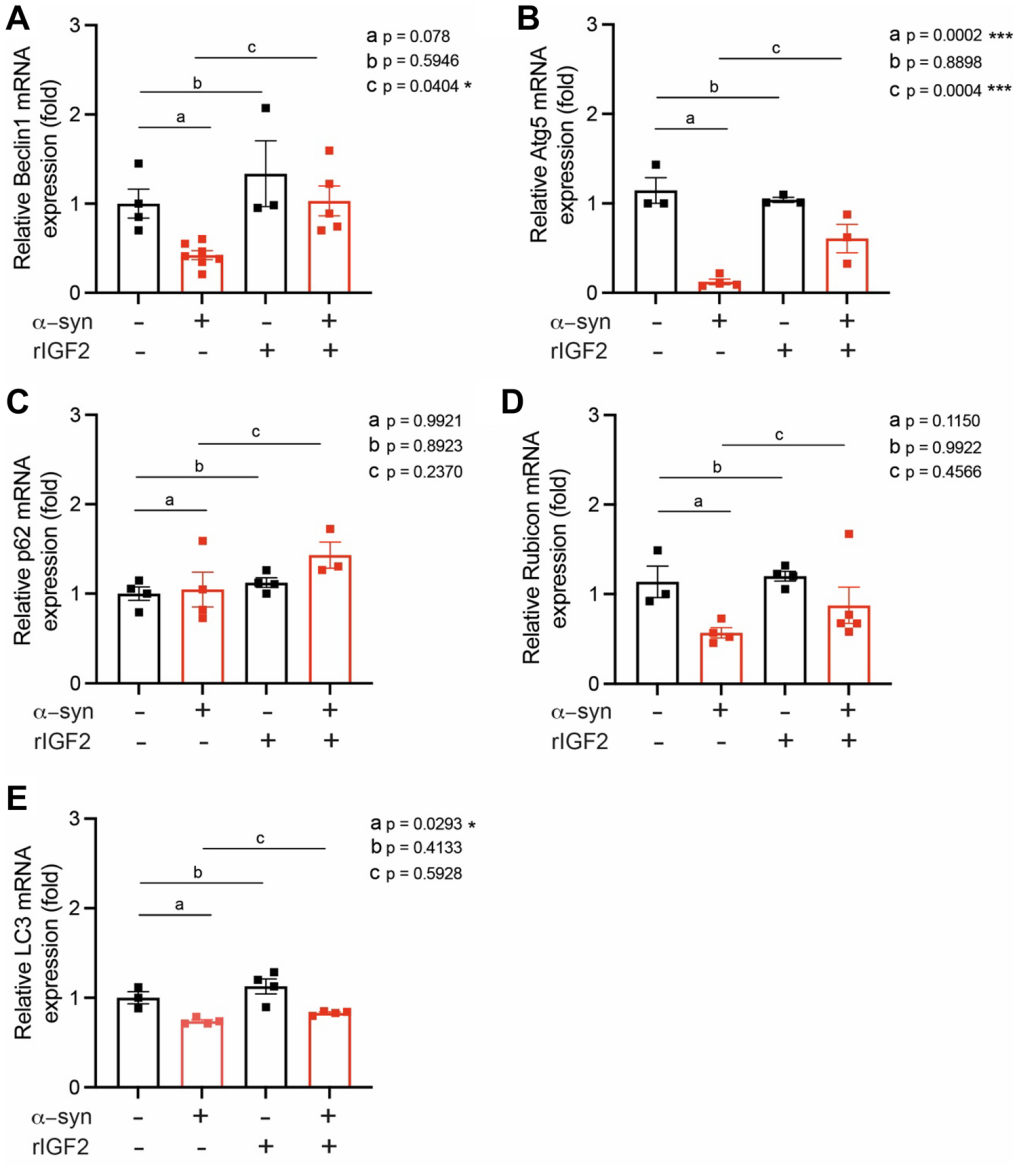
Autophagy genes expression under α-synuclein and IGF2 treatment in macrophages. mRNA levels from (**A**) *Beclin1*, (**B**) *Atg5*, (**C**) *p62*, (**D**) *Rubicon*, and (**E**) *LC3* were measured by real-time PCR in macrophages cultures obtained from bone marrow from ASO mice treated with recombinant IGF2 (rIGF2) and/or α-synuclein PFF or PBS as a control for 72 h. Autophagy-related gene mRNA levels were quantified and normalized to *Actin* mRNA levels. Statistically significant differences were detected by ordinary one-way ANOVA (**p < 0.01; *p < 0.05).

The increased copy number or mutations in the *SCNA* gene encoding α-synuclein are among the causes of PD cases, while the accumulation of α-synuclein proteins is a neuropathological hallmark of all cases of PD^8,9^. It was shown that iPSC-derived macrophages treated under elevated wild-type α-synuclein environment develop a proinflammatory profile, impairing their phagocytosis ability, contributing to PD pathogenesis progression^75^. To evaluate the IGF2 effect on autophagy genes in a PD context, we prepared primary bone marrow macrophages cultures from ASO mice stimulated with wild-type α-synuclein preformed fibrils. ASO mice express human α-synuclein in the CNS, but macrophages from these mice are sensitized to an inflammatory response, emulating the PD systemic context in patients^68^. Macrophages were treated with recombinant IGF2 (5 ng/mL) for 1, 3, and 5 days. After the IGF2 treatment, macrophages were stimulated with α-synuclein preformed fibrils or PBS for 72 h. We then evaluated a similar panel of autophagy genes (Fig. 5) previously determined in PBMCs from HC and PD patients (Fig. 3). We observed that α-synuclein treatment caused a tendency to decrease *Beclin1* levels in macrophages (p = 0.07) (Fig. 5A). However, α-synuclein and IGF2 treatment increased *Beclin1* expression levels significantly compared to macrophages not treated with IGF2 (p < 0.05) (Fig. 5A). Notably, in the case of *Atg5* transcriptional expression, we observed a reduction under α-synuclein treatment, which was reversed by treating with IGF2 (p < 0.001) (Fig. 5B). Levels of *p62* or *Rubicon* were unchanged in any condition (Fig. 5C,D). *LC3B* mRNA levels decreased under α-synuclein treatment alone (p < 0.05), with no differences in cells under IGF2 treatment (Fig. 5E). Thus, our results show a positive link between IGF2 treatment and the expression of two key genes from the initial steps of the autophagy pathway in the cellular PD model.

## Discussion and conclusion

Currently, extensive evidence supports the contribution of autophagy pathway disturbances in the pathogenesis of PD^76–81^. In particular, both α-synuclein and LRKK2, proteins present in Lewy Bodies, have been described as CMA substrates^59,79^. Additionally, alteration of autophagy and CMA has been reported in PD postmortem brain tissues due to the accumulation of misfolded proteins such as α-synuclein in dopaminergic neurons^60,82,83^. However, fewer reports are available describing autophagy impairment in blood cells from PD patients and the possible mirror effect of proteostasis imbalance phenomenon observed in brain tissue. In addition, few reported on IGF2 signaling and its relationship with autophagy impairment in blood cells. Evaluating autophagy gene expression and its correlation with IGF2 levels in samples obtained by non-invasive methods, such as in blood samples, could contribute to an advance in finding a biomarker or predictive markers of the PD.

IGF2 signal transduction and function are highly dependent on the type of target cells. For instance, in pancreatic β cells, the overexpression of IGF2 disrupted islet structure, promoting islet hyperplasia and a pre-diabetic state in mice. At a cellular level, IGF2 overexpression caused increased ER stress, autophagy activation, and β cells dedifferentiation^55,84^. In osteosarcoma^85^ and colorectal cancer cells^54^, IGF2 is upregulated and is associated with autophagy activity induction, potentiating tumor growth. In embryonic skeletal muscles, IGF2R acts as an IGF2 negative regulator and induces IGF2 degradation through lysosomes, modulating the systemic IGF2 levels^86,87^. Indeed, IGF2 plays essential roles in the development process of fast myofibers in youth and aging^88,89^.

On the other hand, our data and other works show that IGF2 overexpression in different brain disease contexts has a neuroprotective effect^33,44,45,90^. In addition, a recent study showed that the systemic treatment of an autism mouse model with IGF2 reverted the negative social behaviors characteristic of autism spectrum disorder^84^. These improvements were associated with reversing abnormal levels of the AMPK-mTOR-S6K pathway in hippocampus samples^84^. One of the most significant inhibitory kinases of autophagy activity, mTOR, links IGF2 functions with the autophagy pathway. In the PD context, a study using the pharmacologic 1-methyl-4-phenylpyridinium (MPP +) neurotoxicity in cell cultures and mice nigrostriatal dopaminergic neurons found neuroprotection by IGF2 related to antioxidant effects and improvement of mitochondrial function^19^. Moreover, an increase in the mTOR phosphorylation in cell culture treated with IGF2 has been reported^50^. However, the relationship between IGF2 and autophagy activity was not evaluated in this work.

In the present research, we found a stronger decreased mRNA and protein of IGF2 levels in PBMCs from PD patients, suggesting a relationship between the downregulation of IGF2 and neurodegenerative pathology. Regarding IGF1 levels, we do not observe changes in the plasma samples, which is a controversial result considering previous works showing elevated IGF1 levels in serum of PD patients^28,29^.

Additionally, in the same patient cohorts, we report a downregulation of genes involved in the initial steps of the autophagy pathway, *ULK1*, *BECLIN1, and ATG5*. Of note, changes in the expression of these genes in blood samples from AD patients were also identified as contributing to disease pathogenesis in different studies^91^. ULK1 engages the initiation of autophagosome formation induced by amino acid deprivation, accumulation of protein aggregates, or under mitophagy activation (selective autophagy of dysfunctional mitochondria)^92,93^. BECLIN1 is a protein present in at least two pathways’ complexes from the autophagy pathway, acting as a positive member of the class III PI3K^94^. ATG5 is a protein essential for forming autophagosomes, acting in a complex with ATG12 and ATG16L, downstream from the ULK1 complex^95^. The reduction of these genes from initial pathway complexes suggests a decrease in the formation of the autophagosome vesicles that surround the substrate. Recently, an ultrastructural study in PBMCs obtained from PD patients demonstrated fewer autophagy vacuoles per cell, supporting our results^96^. In particular, we found that the reduction of *BECLIN1* and *ATG5* in PBMCs of PD patients was aligned with the significant reduction in transcriptional levels of *Atg5* and a tendency in *Beclin1* in murine macrophages treated with α-synuclein. Notably, the incubation of these cells with recombinant IGF2 reverted the α-synuclein effect, increasing the transcriptional levels of *Beclin1* and *Atg5*. Recently, an SNP in the gene encoding ATG5 was reported to increase the susceptibility to PD with cognitive impairment in Chinese patients^97^. This SNP results in reduced ATG5 plasma levels in early-onset PD patients, proposing a role for ATG5 deficiency in PD pathology. In this line, ATG5-dependent autophagy mediates the degradation of NLRP3 (NLR family, pyrin domain containing 3) inflammasomes, which can contribute to the pathogenesis in PD^98^. Another study has recently shown a link between increased levels of a miRNA (micro-RNA) molecule, miR-30c-5p, and reduced levels of ATG5 in PD patients^99^. By using an antagonist of this miR-30c-5p, researchers were able to restore Atg5 levels in a murine PD model, attenuating apoptosis in brain tissues^99^. Another work, using zebrafish treated with MPTP as an organism model of PD, showed that the overexpression of Atg5 rescued dopaminergic neuronal loss and autophagy activity^100^. Altogether, our results propose a reduction in IGF2 and autophagy genes from the initial stages of the autophagy pathway as genetic changes in blood samples of PD patients. In particular, our cell culture results strengthen evidence of a correlation between IGF2 levels and *Beclin1* and *Atg5* transcriptional regulation, proposing a protective effect of IGF2 on the autophagy pathway in macrophages.

We also evaluated a potential gender difference in the expression of autophagy genes in patients. It is known that PD is more prevalent in men than women, including presenting differences in severity and progression, and these differences are not entirely understood. A study of the gene expression profile of dopaminergic neurons from substantia nigra pars compacta in men and women found an increased expression of α-synuclein in men^101^, which could partially explain the prevalence of PD in males. In our study, female PD patients strongly correlated with reduced *IGF2* and *BECLIN1* mRNA levels. A possible explanation of these differences could be hormone levels. Of note, 17β-estradiol were shown to influence autophagy (Coto-Montes et al., 2009), while Beclin1 was reported to downregulate 17β-estradiol signaling, proposing an unexplored interaction between the two pathways (Cahill, 2006; Cantuti-Castelvetri et al., 2007). Interestingly, the *ATG5* expression profile was more correlated to male PD patients, potentially contributing to the increased prevalence of PD in males.

The effect of IGF2 signaling on PD and its relationship to the autophagy pathway needs further research. However, a recent study showed that IGF2R deletion in cervical cancer cells disrupted the Golgi-to-lysosome transport of M6P-tagged cathepsins, decreasing lysosomal activity, impairing autophagy and mitophagy activity^102^. It has also been reported that there is a reduction of lysosomal activity in PBMCs cells from PD patients^103^. These data allow a hypothesis that deficient levels of IGF2 reduce hydrolases transport to the lysosomal lumen, generating negative feedback on autophagy genes from the initial stages of autophagy and mitophagy pathways to cope with lysosomal failure. This theory aligns with the increased levels of *RUBICON*, an inhibitory protein from the autophagosome maturation^104,105^, observed in PBMCs from PD. IGF2 treatment should restore lysosomes luminal hydrolases and initiate autophagosome formation, observed by increased *Beclin1* and *Atg5* in macrophage cell culture treated with IGF2. Interestingly, in macrophages treated with α-synuclein, we observed almost opposite results on *Rubicon* levels. PBMCs are most represented by lymphocytes, with about 10% phagocytic macrophages. Besides being a negative regulator of the autophagy pathway, Rubicon is an essential protein for the LC3-associated phagocytosis (LAP), active in phagocytic cells as the macrophages^106^. In this process, phagocytic cells invaginate extracellular cargos via a uni-membrane vesicle dependent on LC3B, Rubicon, and Atg5^106^. In PBMCs, the increased *RUBICON* levels indicated an effect on autophagy activity, i.e., a reduction in autophagy activity. However, in isolated macrophages, phagocytic cells, Rubicon and LC3B changes could result in autophagy and/or LAP activities stimulations. This hypothesis and the pathway activity priority in PBMCs and macrophages in a PD context should be further tested using PD animal models or cell-specific culture exposed to α-synuclein.

The IGF2 protective effect on CNS phenotypes in PD and its mechanism on autophagy activity needs to be further evaluated in animal models of PD, including both genders, during the progression of the disease.

Taken together, our results suggest a change of the proteostasis network associated with reducing IGF2 and autophagy in PD patients. Our findings can contribute to elucidating the potential of IGF2 and the autophagy process on PD's pathogenesis and their possible use as multi-predictor biomarkers for PD early diagnosis and/or progression, also considering the gender of the patients. Moreover, IGF2 appears as a protective agent mediating autophagy activity, becoming a therapeutic molecule of interest for PD and other neurodegenerative diseases.

## Supplementary Information

Supplementary Information.
