# Supplementary material for: Insulin-like growth factor 2 and autophagy gene expression alteration arise as potential biomarkers in Parkinson’s disease
Source: Sci Rep. 2022 Feb 7;12:2038. doi: 10.1038/s41598-022-05941-1 (PMC8821705; doi:10.1038/s41598-022-05941-1)
Supplement: Supplementary file 1 — Supplementary Information. [file 41598_2022_5941_MOESM1_ESM.docx]

**Supplementary Fig. S1:** Raw data for Figure 1C. IGF2 WB of total protein extracts isolated from peripheral blood mononuclear cells (PBMCs) of PD patients and HC subjects. GADPH and actin was determined as a loading control.

**Supplementary Fig. S2:** Housekeeping gene analysis in PD patients. *IGF2* mRNA levels were measured and normalized to *SHDA* mRNA levels by real-time PCR in PBMCs obtained from PD patients and HC subjects. (PD, N=6; HC, N=6). Statistically significant differences were detected by two-tailed unpaired *t*-test (**: *p* <0.01).

**Supplementary Fig. S3:** Gender analysis of Autophagy components in PBMCs from PD patients. mRNA levels were measured by real-time PCR in PBMCs obtained from PD patients separated by gender. (Women patients, black square, N=20; men PD patients, red square, N=21). Autophagy-related gene mRNA levels were quantified and normalized to *SHDA* mRNA levels.
